# Short- and Long-Term Evaluation of Renal Function after Radical Cystectomy and Cutaneous Ureterostomy in High-Risk Patients

**DOI:** 10.3390/jcm9072191

**Published:** 2020-07-11

**Authors:** Massimiliano Creta, Ferdinando Fusco, Roberto La Rocca, Marco Capece, Giuseppe Celentano, Ciro Imbimbo, Vittorio Imperatore, Luigi Russo, Francesco Mangiapia, Vincenzo Mirone, Domenico Russo, Nicola Longo

**Affiliations:** 1 Urologic Section, Department of Neurosciences, Sciences of Reproduction, and Odontostomatology, University of Naples Federico II, 80131 Naples, Italy; robertolarocca87@gmail.com (R.L.R.); drmarcocapece@gmail.com (M.C.); dr.giuseppecelentano@gmail.com (G.C.); ciro.imbimbo@unina.it (C.I.); mangiapippo@libero.it (F.M.); mirone@unina.it (V.M.); nicolalongo20@yahoo.it (N.L.); 2 Department of Urology, Luigi Vanvitelli University of Naples, 80131 Naples, Italy; ferdinando-fusco@libero.it; 3 Urology Unit, Buon Consiglio Fatebenefratelli Hospital, 80123 Naples, Italy; v.imperatore@alice.it; 4 Nephrology Unit, Ospedale del Mare; 80131 Naples, Italy; luigirusso82@hotmail.it; 5 Nephrology Unit, Department of Public Health; University of Naples Federico II, 80131 Naples, Italy; domenico.russo2@unina.it

**Keywords:** renal function, acute kidney injury, GFR deterioration, bladder cancer, radical cystectomy, cutaneous ureterostomy

## Abstract

Deterioration of renal function has been reported after radical cystectomy (RC) with urinary diversion. We investigated renal function changes in elderly bladder cancer (BCa) patients who underwent RC with cutaneous ureterostomy (CU) urinary diversion. We performed a retrospective, observational study. BCa patients aged ≥75 with an American Society of Anesthesiologists (ASA) class greater than II were included. Glomerular filtration rate (GFR) was the main outcome measure. GFR values were recorded preoperatively, at discharge, at 6-month follow-up, and yearly up to 60 months. A total of 70 patients with a median age of 78.0 years were identified. Median preoperative GFR was 74.3 mL/min/1.73 m^2^ and declined significantly to 54.6 mL/min/1.73 m^2^ after 6 months (*p* < 0.001). A gradual GFR decline was observed thereafter, reaching a median value of 46.2 after 60 months. Preoperative GFR and acute kidney injury were significant predictors of fast deterioration of GFR and of 25% deterioration of GFR after 12 months. Elderly BCa patients with high comorbidity rates undergoing RC with CU should be carefully informed about the risk of GFR deterioration and the need for adequate monitoring.

## 1. Introduction

Radical cystectomy (RC) with pelvic lymph node dissection and urinary diversion represents the standard of care for patients with muscle-invasive bladder cancer (BCa) and for selected patients with high-risk, nonmuscle-invasive disease [1,2,3].

This surgical procedure has been reported to be associated with deterioration of renal function with subsequent increased risk of perioperative and long-term morbidity and mortality due to the established association between chronic kidney disease (CKD) and cardiovascular morbidity and mortality [3,4,5,6,7]. Therefore, careful nephrological evaluation is required in patients who have undergone RC with urinary diversion.

To date, only a few studies have investigated the changes in renal function in this subset of patients, providing controversial results [4,7,8,9]. Several potential limitations hamper the results of previous studies. Indeed, changes in renal function were evaluated, comparing various surgical procedures such as continent and incontinent diversions, and criteria adopted to score renal function were heterogeneous. Furthermore, patients were followed up at very large intervals [10]. Finally, data on acute and year-by-year changes in renal function after RC with urinary diversion are very scarce.

The aims of the present study were (1) to investigate the acute and year-by-year changes in renal function in a population of elderly patients with high rates of comorbidities who had undergone RC with cutaneous ureterostomy (CU) as the sole urinary diversion procedure and (2) to evaluate the changes in renal function as suggested by international guidelines.

## 2. Material and Methods

We performed a retrospective, observational study. Inclusion criteria were BCa patients with a history of RC with CU urinary diversion performed at a single institution, age ≥75 years, and American Society of Anesthesiologists (ASA) class greater than II. Exclusion criteria were palliative cystectomy for massive bleeding, presence of distant metastases at the diagnosis, concomitant upper urinary tract transitional cell carcinoma, simultaneous urethrectomy, follow-up <1 year, and insufficient data. All patients underwent open RC using standard techniques [9]. The surgical procedure for CU has been described previously [1]. Patients were seen in the outpatient clinic every 3 months for the first year, every 6 months for the next year, and on a yearly basis thereafter. Follow-up consisted of a history with a physical examination, a biochemical profile, and ultrasonography of the abdomen. Computed tomography of the chest and abdomen was required every 6 months until the third year, followed by annual imaging thereafter. Age, gender, body mass index (BMI), comorbidities, ASA class, presence of preoperative hydronephrosis, pathological stage, neoadjuvant or adjuvant chemotherapy, and episodes of postoperative pyelonephritis were recorded. Patients with systolic blood pressure >140 mmHg and/or diastolic blood pressure >90 mmHg were regarded as hypertensive. Diabetics were defined as patients undergoing chronic therapy with hypoglycemic drugs. BMI was calculated by dividing weight in kilograms by the square of the patient’s height in meters.

The age-adjusted Charlson Comorbidity Index (CCI) was used to define preoperative comorbidity [11].

The primary outcome measure was glomerular filtration rate (GFR). It was estimated with the modification of diet in renal disease (MDRD) equation, eGFR (mL/min/1.73 m^2^) = 175 × (serum creatinine) − 1.154 × (age) − 0.203 × (0.742, if female) and CKD stages were used to further classify renal function [12,13,14,15]. GFR was recorded before surgery; at discharge; and 6, 12, 24, 36, 48, and 60 months after surgery. Deterioration of renal function over time was evaluated based on a certain drop in GFR category accompanied by a 25% or greater drop in GFR from baseline [16]. Patients with fast deterioration due to CKD were identified by loss of GFR by ≥5 mL/min/1.73 m^2^/year [17]. Acute kidney injury (AKI) was characterized by an acute increase in serum creatinine concentration within 4 days based on the following criteria for CKD stages: CKD stage I = an increase in serum creatinine of greater than or equal to 0.3 mg/dL or an increase of greater than or equal to 150% to 200% (1.5- to 2-fold) from baseline; CKD stage II = an increase in serum creatinine of more than 200% to 300% (>2- to 3-fold) from baseline; CKD stage III = an increase in serum creatinine of more than 300% (>3-fold) from baseline (or a serum creatinine level of greater than or equal to 4.0 mg/dL with an acute increase of at least 0.5 mg/dL [16,18].

Patients’ data were analyzed as a whole cohort as well as by dividing patients according to lateralization of stoma (unilateral or bilateral) and presence of diabetes.

Continuous variables were expressed as median and interquartile range (IQR). The difference between median values was statistically tested using the Wilcoxon test. Categorical variables were compared using the chi-square test or the Fisher test. Multiple linear regression analyses were performed to identify significant preoperative predictors of postoperative deterioration of renal function, taking into account the following potential confounders: age, preoperative GFR, AKI, diabetes, CCI score, ASA class, hypertension, hydronephrosis, and preoperative chemotherapy. *p* values < 0.05 indicated statistical significance. Statistical analyses were performed using SPSS version 17.0 (SPSS Inc., Chicago, IL, USA) software. The study was conducted in accordance with the Declaration of Helsinki of 1975, revised in 2013. Patients were retrospectively identified from a database approved by University of Naples Federico II Institutional Review Board (#8914). All patients gave their informed consent to the anonymous collection of clinical data in the database and to the use of these data for research purposes.

## 3. Results

A total of 70 patients who underwent RC with CU urinary diversion at our institution between March 2012 and March 2017 were identified. Of these, 41 (58.6%) and 29 (41.4%) underwent unilateral and bilateral stoma CU, respectively. Surgical procedures were performed by the same surgical team. The median (IQR) follow-up after RC was 60.0 (24.0) months.

Demographic and clinical characteristics and some patient laboratory data are reported in Table 1.

Diabetics had a similar median age and BMI to nondiabetics but a lower baseline GFR (IQR) (67.6 (27) vs. 73.7 (38) mL/min; *p* < 0.001).

**Figure 1:**
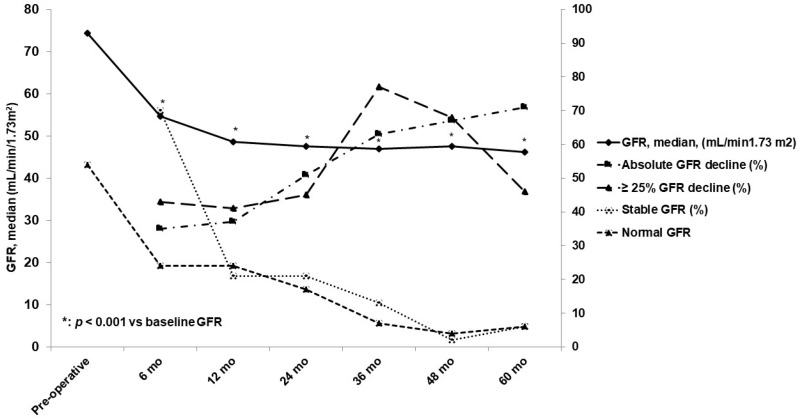
Changes in glomerular filtration rate (GFR) over time in the whole cohort.

Baseline data and changes of kidney function over time in the whole cohort are reported in Table 2 and Figure 1.

Before surgery, many patients were categorized as having CKD stage I–IIIA. GFR progressively declined after surgery. The mean decline of GFR at 6 months was 20 mL/min (35% lower than basal value); this decline was confirmed at the 12-month follow-up (mean decline of 21 mL/min; 37% lower than basal value). Subsequently, a gradual decline in GFR was recorded from the 36th month to the 60th month. Opposite to the trend in GFR, the percentage of patients showing a decline in GFR by ≥25% increased from the 24th month to the 48th month. Seventy percent of patients had stable or improved GFR at 6 months. Stable or improved GFR was more frequent in males and in patients with a pre-surgery GFR ≥50 mL/min and ASA class III or lower, but it was less frequent in patients with diabetes, a CCI score ≥7, hydronephrosis, and urinary tract infections. Pre-surgery GFR was a unique, significant predictor (*p* < 0.01). The percentage of patients with stable or improved GFR was much lower early after surgery. The trend of GFR variation in patients who had undergone unilateral CU was similar to that seen in patients who had undergone bilateral CU. The post-operative trend of median GFR in patients following unilateral stoma was similar to the one observed in the whole cohort. In contrast, patients who underwent bilateral CU had significantly lower GFRs at baseline and at each post-surgery follow-up point compared to patients who underwent unilateral stoma (Table 3). No statistically significant differences were observed between patients with and without diabetes in terms of GFR decline. No AKI was recorded in diabetics.

GFR at discharge was available for a subgroup of 39 patients, enabling us to determine earlier changes in renal function. The clinical characteristics of this subgroup included a median age of 77.0 years (IQR = 4.0); 90% were male; 18% had diabetes; 51% had hypertension; and 39% underwent bilateral stoma. Median (IQR) GFR declined by 15% from 74.5 (36.0) to 67.3 (25.0) mL/min (*p* < 0.01); worsening of renal function was evidenced in 31% of patients. AKI was registered in seven patients (18%). In a stepwise multiple linear regression analysis, among the potential confounders taken into account, preoperative GFR and AKI were significant predictors of fast deterioration of GFR (*p* < 0.02) and of 25% deterioration of GFR (*p* < 0.02) at 12 months.

## 4. Discussion

RC with urinary diversion in BCa patients represents a surgical procedure potentially associated with deterioration of renal function [19]. In turn, impaired renal function can compromise cardiovascular health, mainly in older and frail populations such as BCa patients. The natural history of renal function after RC with urinary diversion is under-reported, and the results are often contradictory [4,7,8,9,10]. Relevant limitations include the criteria adopted to measure the decline in renal function and the time points of follow-up. Indeed, estimation of renal function based only on serum creatinine concentration is misleading due to sarcopenia in old and frail people [10,12,14]. The measurement of serum creatinine concentrations does not allow for the evaluation of long-term changes in renal function, which can be influenced by variations in nutritional status. However, scoring the level of renal function based only on CKD stage is affected by the width range of GFR, primarily in the II–III CKD stages, where a decrease in GFR of more than 10 mL/min is considered stable but would be considered worsened if the patient were in CKD stage IV [10]. Using the decrease in GFR by 1 or 3 mL/min per year or an absolute decrease of ≥25% from baseline many years later (as far as 10 years) in a unique post-surgery record as a marker of renal function does not take into account, on the one hand, the age-dependent decline of GFR by 1 mL/min per year that is regarded as physiological or, on the other hand, year-by-year changes in renal function due to intercurrent events during a long observational period [15]. Indeed, patients who undergo RC with urinary diversion are exposed to many risk factors. Chemotherapy, surgical distress, urinary infection, pyelonephritis, nephrotoxic medications, and urinary tract obstruction may all negatively affect renal function [4,7,8,20,21,22,23,24].

In a previous study, we evaluated complications and quality of life in elderly patients with high comorbidity status who had undergone either an ileal conduit or a CU urinary diversion, and we found more relevant advantages to CU compared to ileal conduit such as reduced duration of surgery, reduced bleeding, and no need for bowel resection, with a consequently lower incidence rate of complications and mortality [1]. Therefore, CU might be considered a preferable urinary diversion after RC, particularly in elderly patients at high surgical risk. This position has been shared by others [25].

However, little evidence exists regarding renal function after RC with CU in this subset of patients [4,23]. In the present study, we evaluated, for the first time, the changes in renal function in a cohort of elderly BCa patients at high surgical risk due to high comorbidity status undergoing RC with CU urinary diversion. A further unique characteristic of the present study is the evaluation of renal function across a long post-surgery phase, with year-by-year data recorded according to indications listed in international guidelines [15].

We found a statistically significant decline in GFR after surgery. This finding is in line with published evidence. Indeed, studies have reported the occurrence of renal function decline in approximately 70% of patients undergoing RC with urinary diversion [23].

The changes in GFR recorded in the present study at the 5-year follow-up are in line with those reported at similar follow-up times in patients who underwent other types of urinary diversions, such as ileal conduit and ileal neobladder, that carry more relevant surgical risks [4,7,8,20,23,24].

Different trends in decline have been reported. Makino et al. described a rapid GFR decline during the first year after RC (−6 mL/min/1.73 m^2^) and a slow, continuous decrease in subsequent years (about 1 mL/min/1.73 m^2^ per year) [3]. Similarly, Rouanne et al. observed a rapid GFR decline during the first 2 years after surgery (−9 mL/min/1.73 m^2^ in the first year and −4 mL/min/1.73 m^2^ in the second year) followed by a slow decrease in the subsequent year [7]. Unlike previous authors, Nishikawa et al. found a monophasic postoperative decline in GFR [19]. Our results confirm the occurrence of a biphasic deterioration of GFR in these patients that was characterized by a rapid decline during the first 6 postoperative months and a gradual decrease in subsequent years. Interestingly, we found a high percentage of patients (70%) with stable or improved GFR at the 6-month follow-up, and the percentage of reduction in GFR both as a decrease of ≥25% and as an absolute percentage from baseline was more relevant in later post-surgery phases. Although we can hypothesize that the relief of upper urinary tract obstruction may have contributed to the improvement in GFR observed early after RC with CU, this pathophysiological mechanism may be true only for a small percentage of patients in the present series. Of note, stable and/or improved GFR was observed at 6 months in males, in patients with a pre-surgery GFR ≥50 mL/min, and in patients belonging to ASA class III or lower. These data indicate that better pre-surgery renal function and fewer comorbidities may play a role in the rate of GFR decline, at least during the early post-surgery phase. However, further studies are needed to confirm and better clarify the rationale behind these observations. The etiology of postoperative GFR decline is multifactorial, and several nonmodifiable or modifiable risk factors have been found as predictors, such as age, preoperative GFR, chronic hypertension, diabetes mellitus, preoperative hydronephrosis, postoperative pyelonephritis, postoperative urinary tract obstruction, and chemotherapy [4,7,8,20,21,22,23]. In the present study, only preoperative GFR and AKI were significant predictors of fast GFR deterioration and of 25% GFR deterioration at the 12-month follow-up. Currently, there is no evidence comparing postoperative renal function between patients who underwent unilateral CU and patients who underwent bilateral CU. We found that the trend in GFR decline was independent from the type of CU urinary diversion. Likewise, there was no difference in percentage of patients showing either reduction in GFR by ≥25% or fast deterioration of GFR. Interestingly, the rate of stable or improved GFR after surgery was lower in patients who had undergone bilateral stoma. The rationale behind this observation remains controversial and deserves further research. The role of diabetes is controversial as it has been reported as a predictive factor in some studies but not in others [2,4,7,20,21,23,24]. Results from the present study failed to find differences in worsening GFR between patients with and without diabetes. Data of clinical relevance were attained when earlier changes in renal function were evaluated in this study. At discharge, GFR was already significantly reduced; the median decrease in GFR was 15%, and 31 patients had worsened renal function. There were seven cases of AKI (18%), but none required dialysis treatment.

To the best of our knowledge, we evaluated for the first time the natural course of renal function in elderly BCa patients with high comorbidity status who had undergone RC with CU urinary diversion. Although the inclusion of patients with these features may potentially bias the results by overestimating eGFR decline following surgery, this study population is highly representative of RC with CU urinary diversion candidates encountered in everyday clinical practice and represents a subset of patients whose epidemiological relevance will increase in the coming years.

Despite efforts to address potential sources of bias (e.g., monocentric design with involvement of the same surgical team, exclusion of patients with concomitant upper urinary tract transitional cell carcinoma requiring concomitant nephroureterectomy), our study is mainly limited by its retrospective, nonrandomized design. The small sample size represents a further limitation of the study. However, it should be emphasized that our series represents one of the largest published to date involving elderly BCa patients with multiple comorbidities. Furthermore, the lack of a control cohort that did not undergo surgery to assess the comparative impact of RC with CU urinary diversion on renal function decline represents, as in the majority of studies on the same issue, a methodological limitation. However, the identification of a comparison group in this clinical scenario is challenging. In fact, it should be noted that BCa itself may alter renal function, and a control cohort of patients without BCa would not allow us to adequately investigate the effects of surgery on renal function. On the other hand, utilizing a control group of elderly BCa patients with high comorbidity status fit for RC with CU would not be ethical. Other potential limitations of the study include unavailability of data about concomitant medical therapies, smoking habits, nutritional conditions, and levels of proteinuria, as well as the absence of patients who had undergone preoperative chemotherapy. Consequently, our data should be considered preliminary, and further, well-designed studies involving large cohorts are needed to improve the level of knowledge about the natural history of renal function in this subset of BCa patients.

## 5. Conclusions

Results from the present study provide information about changes in renal function occurring after RC with CU in elderly BCa patients at high surgical risk, a subset of patients often encountered in everyday clinical practice but poorly represented in current literature. A biphasic variation of renal function was observed, characterized by a rapid, early decline followed by a gradual one. Based on the results from the present study, elderly patients with high comorbidity status who are candidates for RC with CU should be carefully informed about the potential risk of both early and late deterioration of renal function as well as about the need for adequate monitoring of renal function, mainly in the first months after surgery when renal function impairment may be more frequent and prompt correction of modifiable risk factors such as electrolyte imbalance, antimicrobial drug nephrotoxicity, and alterations in mineral metabolism may be most beneficial for patients.

## Figures and Tables

**Table 1:** Demographic and some clinical characteristics of the whole cohort.

Age, years, median (IQR)	78.0 (4.0)
Male gender, *n* (%)	59 (84.3)
BMI, kg/m^2^, median (IQR)	27.4 (4.8)
CCI, median (IQR)	5.0 (1.0)
Diabetes, *n* (%)	16 (22.9)
Hypertension, *n* (%)	27 (38.6)
Preoperative hydronephrosis, *n* (%)	8 (11.4)
Preoperative GFR, mL/min/1.73 m^2^, median (IQR)	74.3 (42)
ASA class, *n* (%)	
III	67 (95.7)
IV	3 (4.3)
Operating time, min, median (IQR)	135.7 (32.0)
Pathologic stage, *n* (%)	
pT0	2 (2.9)
pTa/is/1	23 (32.9)
pT2	20 (28.6)
pT3	20 (28.6)
pT4	5 (7.1)
pN+	7 (10)
Neoadjuvant chemotherapy, *n* (%)	0 (0)
Adjuvant chemotherapy, *n* (%)	31 (44.3)
Postoperative pyelonephritis, *n* (%)	37 (52.9)

ASA: American Society of Anesthesiologists; BMI: body mass index; CCI: Charlson Comorbidity Index; CKD: chronic kidney disease; GFR: glomerular filtration rate; IQR: interquartile range.

**Table 2:** Preoperative and follow-up renal function in the whole cohort.

	Baseline *n* = 70	6 mo *n* = 70	12 mo *n* = 70	24 mo *n* = 64	36 mo *n* = 55	48 mo *n* = 48	60 mo *n* = 44
GFR, mL/min, median (IQR)	74.3 (42.0)	54.6 (19.0) *	48.6 (19) *	47.5 (17.0) *	46.9 (22.0) *	47.6 (22.0) *	46.2 (17.0) *
GFR decline from baseline, %	-	35	37	51	63	67	71
GFR decline ≥25% from baseline, %	-	43	41	45	77	68	46
Stable GFR, %	-	52.9	5.7	7.6	3.9	0.7	3.9
Improved GFR, %		17.1	15.3	13.4	9.1	1.3	2.1
Fast GFR deterioration, %	-	-	67	26	19	19	13
CKD stage I, %	25.7	8.6	12.9	4.6	3.6	4.2	2.3
CKD stage II, %	37.1	28.6	13.7	17.1	12.7	14.6	13.6
CKD stage III A, %	30	37.1	41.4	42.7	41.8	23.3	41
CKD stage III B, %	4.3	20.0	24.3	12.5	20.0	27.1	20.4
CKD stage IV, %	0	4.3	4.3	20.3	20.0	18.8	20.4
CKD stage V, %	2.9	1.4	1.4	1.5	1.8	2.1	2.3
Lost to follow-up, *n*	0	0	0	6	15	22	26

* *p* < 0.001 vs. baseline GFR; CKD: chronic kidney disease; GFR: glomerular filtration rate; IQR: interquartile range; mo: months after surgery.

**Table 3:** Baseline and follow-up renal function stratified according to cutaneous ureterostomy technique.

	Unilateral CU					Bilateral CU				
	GFR, mL/min, Median (IQR)	GFR Decline from Baseline, %	GFR Decline ≥25% from Baseline, %	Stable or Improved GFR, %	Fast GFR Deterioration, %	GFR, mL/min, Median (IQR)	GFR Decline from Baseline, %	GFR Decline ≥25% from Baseline, %	Stable or Improved GFR, %	Fast GFR Deterioration, %
Baseline	74.3 (24.8)	-	-	-	-	72.6 (31.0) ^b^	-	-	-	-
6 mo	55.7 (22.0) *	25	43	73	-	51.2 (21.0) *	17	45	67	-
12 mo	52.6 (16.0) *	26	34	22	66	45.4 (15.0) *	22	52	22	69
24 mo	51.4 (22.0) *	29	37	27	22	45.9 (20.0) *^,b^	20	59	15	31
36 mo	48.7 (17.0) *	35	39	17	17	42.1 (22.0) *^,b^	35	48	8	21
48 mo	49.7 (20.0) *	37	41	2	17	40.4 (20.0) *^,b^	25	52	0	21
60 mo	46.7 (16.0) *	37	39	10	17	39.8 (27) *^.b^	35	59	0	7

* *p* < 0.001 vs. baseline GFR; ^b^ *p* < 0.001 bilateral vs. unilateral; CKD: chronic kidney disease; CU: cutaneous ureterostomy; GFR: glomerular filtration rate; IQR: interquartile range; mo: months after surgery.
